# Identifying persistent high-cost patients in the hospital for care management: development and validation of prediction models

**DOI:** 10.1186/s12913-024-11936-7

**Published:** 2024-11-26

**Authors:** Ursula W. de Ruijter, Z. L. Rana Kaplan, Frank Eijkenaar, Carolien C. H. M. Maas, Agnes van der Heide, Willem A. Bax, Hester F. Lingsma

**Affiliations:** 1 Department of Public Health, Erasmus MC University Medical Center, Rotterdam, The Netherlands; 2 Department of Internal Medicine, Northwest Clinics, Alkmaar, The Netherlands; 3 Erasmus School of Health Policy & Management, Erasmus University, Rotterdam, The Netherlands

**Keywords:** Prognosis, Meaningful use, Health expenditures, Managed care programs, Value-based health care

## Abstract

**Background:**

Healthcare use by High-Need High-Cost (HNHC) patients is believed to be modifiable through better coordination of care. To identify patients for care management, a hybrid approach is recommended that combines clinical assessment of need with model-based prediction of cost. Models that predict high healthcare costs persisting over time are relevant but scarce. We aimed to develop and validate two models predicting Persistent High-Cost (PHC) status upon hospital outpatient visit and hospital admission, respectively.

**Methods:**

We performed a retrospective cohort study using claims data from a national health insurer in the Netherlands—a regulated competitive health care system with universal coverage. We created two populations of adults based on their index event in 2016: a first hospital outpatient visit (i.e., outpatient population) or hospital admission (i.e., hospital admission population). Both were divided in a development (January-June) and validation (July-December) cohort. Our outcome of interest, PHC status, was defined as belonging to the top 10% of total annual healthcare costs for three consecutive years after the index event. Predictors were predefined based on an earlier systematic review and collected in the year prior to the index event. Predictor effects were quantified through logistic multivariable regression analysis. To increase usability, we also developed smaller models containing the lowest number of predictors while maintaining comparable performance. This was based on relative predictor importance (Wald χ2). Model performance was evaluated by means of discrimination (C-statistic) and calibration (plots).

**Results:**

In the outpatient development cohort (*n* = 135,558), 2.2% of patients (*n* = 3,016) was PHC. In the hospital admission development cohort (*n* = 24,805), this was 5.8% (*n* = 1,451). Both full models included 27 predictors, while their smaller counterparts had 10 (outpatient model) and 11 predictors (hospital admission model). In the outpatient validation cohort (*n* = 84,009) and hospital admission validation cohort (*n* = 20,768), discrimination was good for full models (C-statistics 0.75; 0.74) and smaller models (C-statistics 0.70; 0.73), while calibration plots indicated that models were well-calibrated.

**Conclusions:**

We developed and validated two models predicting PHC status that demonstrate good discrimination and calibration. Both models are suitable for integration into electronic health records to aid a hybrid case-finding strategy for HNHC care management.

**Supplementary Information:**

The online version contains supplementary material available at 10.1186/s12913-024-11936-7.

## Background

Current delivery of healthcare is disease-oriented and often fragmented, which can be challenging for people with multiple long-term conditions [1, 2]. A small proportion of these patients, referred to as High-Need High-Cost (HNHC), incur a disproportionate share of total healthcare cost [1, 2]. Their complex medical needs often result in potentially preventable healthcare use such as Emergency Room (ER) visits or hospitalization [2, 3]. These high levels of healthcare use are believed to be modifiable through better coordination of care [2, 4]. Additionally, HNHC patients have expressed a desire for improved coordination among their care providers [5, 6]. Patients who remain HNHC over time have become a target for care management in pursuit of the triple aim: enhancing care experiences, improving population health, and containing cost [7–9].

Crucial to achieving these aims is inviting the ‘right’ patients into care management: persistent HNHC patients with modifiable healthcare use, who are willing and able to participate [2, 4, 10]. In designing a case-finding strategy for HNHC care management, no quantitative definition fully encompasses the complexity of HNHC patients [2]. Previous research has therefore recommended a hybrid strategy, combining clinical assessment of care needs (i.e. High-Need) with prediction modelling of healthcare costs (i.e. High-Cost) [4, 11, 12].

Models predicting high costs persisting over multiple years are particularly relevant to the longer time horizon of care management [7, 13]. However, a recent systematic review identified only three non-proprietary models predicting *Persistent* High-Cost (PHC) [13–17]. All three models included predictors that are typically unavailable to clinicians upon use of the model (e.g., prior cost). Furthermore, some models predicted PHC at moments that are less suitable to contemplate inclusion into care management [13–17]. For example, a hectic ER may not offer the same opportunity for shared decision-making on the suitability of care management as an outpatient visit or hospitalization.

The objective of this study is to develop and validate two models predicting future PHC upon a patient’s first outpatient visit to the hospital or upon hospital admission, which are readily applicable as part of a hybrid HNHC case-finding strategy within the hospital.

## Methods

We performed a retrospective cohort study using a claims database obtained from a nationally operating health insurer in the Netherlands. This study is reported according to Transparent Reporting of a multivariable prediction model for Individual Prognosis Or Diagnosis (TRIPOD) guidelines, except for public availability of data [18].

### Setting

As of 2006, the Netherlands has a regulated competitive healthcare system with universal coverage governed by the health insurance act [19]. In this system, insurers compete for enrolees on price and quality of purchased healthcare services and providers compete for favourable contracts with insurers. The basic benefits package covers most curative care, however long-term care (e.g., nursing home care) and preventive care (e.g., vaccination schemes) are covered under different acts [19]. Health insurance coverage is compulsory for all citizens and health insurers must accept all applicants at a community-rated premium. When delivering services under the health insurance act, clinicians record so-called ‘Diagnosis Treatment Combinations’ (DTCs) in the electronic health records (EHRs), similar to Diagnosis Related Groups (DRGs) from other countries [19, 20]. EHRs in the hospital therefore include a current list of DTCs. Non-acute hospital care requires a referral from a general practitioner, who acts as gatekeeper [19]. Specialized outpatient care is primarily provided in hospitals, with only a small fraction in treatment centres outside of the hospital [19]. When registering a DTC, clinicians make a distinction between a first visit to a specific medical department (e.g., cardiology, pulmonology etc.) and subsequent follow-up visits which enables distinguishing between the two in EHRs.

### Outcome and predictors

The definition of our outcome of interest, Persistent High-Cost (PHC) status, was based on three prior studies that were identified in a recent systematic review [14–17]. In these studies, definitions of High-Cost ranged from belonging to the top 1–10% of the cost distribution while *persistent* High-Cost was defined across the studies as being in in the top 1–10% for two to five consecutive years [13]. For the purpose of case-finding for HNHC care management, we wanted to focus on patients with persistently high costs characterised by chronic conditions [21]. Therefore we wanted to move beyond one or two years, while it has been shown that the added benefit of more than three years does not outweigh the prolonged data collection [2, 22]. In our study we therefore defined PHC as belonging to the top 10% of the cost distribution for three consecutive years after the index event, irrespective of healthcare costs in the year prior. As a sensitivity analysis, all analyses were repeated with a High-Cost threshold of top 5%.

Seventy predictors were considered for model building, based on the same systematic review [14–17]. Predictors included demographics, diagnoses, self-rated items, and prior use [13, 14] (Additional file 1). Thirty-two predictors on prior costs were excluded because they would be unavailable to clinicians upon use of the model. A further nine predictors that could not be defined in a universal manner (e.g., survey variables) were excluded. Finally, predictors with an occurrence of ≤ 0.5% at baseline were discarded. All included predictors were operationalized through Clinical Classifications Software Refined (CCSR) codes to facilitate external validation and integration into EHRs. Predictors were collected from a 365-day window preceding the index event. As the data concerned claims data, there was no missingness among predictors.

### Data collection and patient population

We used claims data of a national insurance company in the Netherlands. Claims were limited to those covered under the health insurance act. Costs were scaled to the level of 2015 to account for inflation and ensure comparability across calendar years. Scaling involved multiplying the annual costs of each individual by that year’s scaling factor, which was determined by dividing the median of 2015 by the median of total annual costs in any given year.

**Fig. 1:**
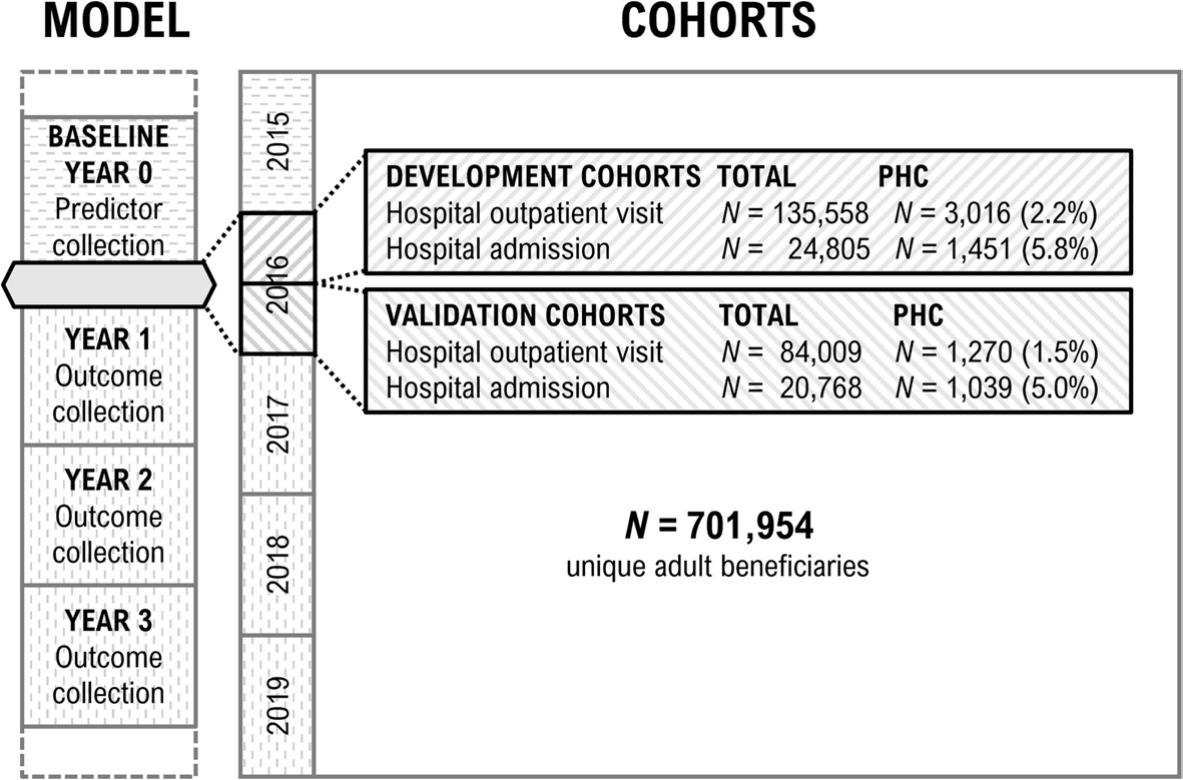
Overview of prediction model structure and establishment of development and validation cohorts over time. PHC = persistent high-cost, defined as belonging to the top 10% of the cost distribution for three consecutive years. All index events (i.e. hospital outpatient visits and hospital admissions) were collected from 2016 and subsequently allocated to a development and validation cohort. Predictors were collected from a 365-day window preceding the index event (i.e. year 0), while outcome was collected in the three years after the index event. The date of the index event was defined as the date of the outpatient visit (hospital outpatient visit population) or the date of first day of an in hospital stay (hospital admission population)

To develop models that predict patients’ PHC status at strategic moments for inclusion into care management, we selected their first outpatient visit to a medical specialist in the hospital or a hospital admission as their index event. The index event was defined by the date of the outpatient visit (hospital outpatient visit population) or the date of first day of an inhospital stay (hospital admission population). Because both index events were collected separately, patients could have entered into both cohorts. To enable predictor collection in the year preceding the index event and outcome collection in the three years after, we required five calendar years of data. However, due to the COVID-19 pandemic we were unable to collect reliable data on healthcare use for 2020 and 2021. Consequently, we analysed claims data from January 1st, 2015, to December 31st, 2019 (Fig. 1).

Two cohorts were created based on patients’ index event in 2016: the hospital outpatient visit cohort and the hospital admission cohort. Patients without consecutive availability of data between January 1st, 2015, and December 31st, 2019, were excluded. In case of disenrollment, no distinction could be made between different reasons for disenrollment, including death. Finally, patients who were under 18 years of age at their index event were excluded considering the different approach to paediatric High-Need High-Cost (HNHC) care management [2].

Cohorts were split for temporal validation purposes. Patients with an index event between January 1st and July 1st, 2016, were used for model development while those with an index event between July 2nd and December 31st, 2016, were used for validation (Fig. 1). If patients had multiple events in 2016, the earliest was used as their index event.

### Model development, validation, and analysis

Predictor effects on the outcome were assessed with logistic multivariable regression analysis and quantified as odds ratios. To gain insight into the relative importance of individual predictors in the two models, the added predictive value of each predictor to the model was quantified using the Wald χ2 test [23]. To further facilitate clinical implementation, parsimonious counterparts were identified for both the outpatient visit and hospital admission model by selecting those predictors with a high relative importance (proportion of χ2 ≥ 0.001) and a *p*-value ≤ 0.2 in the logistic regression analysis. This liberal *p*-value was chosen to prevent overfitting [24–26].

Model performance was evaluated through discrimination and calibration. Discrimination, quantified by the C-statistic, indicates the model’s ability to discriminate between patients with and without the outcome [24, 27]. A C-statistic of 0.5 implies the model is performing no better than random chance, while a C-statistic of 1.0 indicates perfect discrimination. A C-statistic ≥ 0.7 is considered to indicate fair discrimination, whether it is regarded as “good” depends on the specific decision problem and the context of implementation [23]. Calibration, depicted through plots, illustrates the agreement between observed and predicted outcomes in the temporal validation cohort. In case of perfect calibration, the calibration curve has a slope of one and an intercept of zero [24, 27]. Models were validated both internally, to assess reproducibility, and temporally, to assess performance in data differing from the development data in time. The latter is not equal to the external validation of the model on a dataset from a completely different source altogether, but temporal validation is preferable to a random split of the data because it adds an aspect of heterogeneity: time. Internal validation and correction for optimism was performed by a bootstrapping procedure with 2000 replications [28]. To further inform implementation, we calculated sensitivity, specificity and positive predictive value for different risk cut-offs. Statistical analyses were performed in R statistical software (packages dplyr, comorbidity, ggplot2, rms). R code was published as a public repository on GitHub [29]. All R code and its underlying data were checked and verified by two co-authors (RK&CM).

## Results

A total of 986,793 adults had at least one claims record under the health insurance act between January 1st, 2015, and December 31st, 2019, of which 701,954 (71%) were consecutively insured during that period. The top 10% threshold for individual annual healthcare costs was established at €6,572 per year. In 2016, 219,567 patients (31.3%) had a first outpatient visit to a medical specialist in the hospital, while 45,573 patients (6.5%) had a hospital admission (Fig. 1). Patients with an index event between January 1st and July 1st were included for model development, resulting in 135,558 patients in the hospital outpatient visit development cohort and 24,805 in the hospital admission development cohort (Table 1). Within the development cohorts, 2.2% (*n* = 3,016) were Persistent High-Cost (PHC) in the outpatient visit population compared to 5.8% (*n* = 1,451) in the hospital admission population. The median age of patients who became PHC was slightly higher compared to patients who did not become PHC (Table 1).

**Table 1:** Baseline year characteristics for the hospital outpatient visit—and the hospital admission development cohorts

	**Hospital outpatient visit population (*n* = 135,558)**		**Hospital admission population (*n* = 24,805)**	
	**Persistent** **High-Cost**^**a**^ **(*n* = 3,016)**	**Others** **(*n* = 132,542)**	**Persistent** **High-Cost**^**a**^ **(*n* = 1,451)**	**Others** **(*n* = 23,354)**
Sex; *no of females (%)*	1,703 (56.5%)	83,097 (62.7%)	760 (52.4%)	14,148 (60.6%)
Age in years; *median (IQR)*	62 (50—71)	58 (45—69)	63 (53—71)	61 (46—72)
CCI; *median (IQR)*	1 (0—2)	0 (0—0)	1 (1—2)	0 (0—0)
**Healthcare use in the year prior to index event (Y0)** *mean visits per 1000 patients*				
Number of ER visits	155	36	330	124
Number of surgeries	693	261	842	378
Number of inpatient days	4424	741	6379	1590
**Diagnoses** *no (%)*				
Acute cerebrovascular disease	59 (2.0%)	1,414 (1.1%)	37 (2.5%)	421 (1.8%)
Acute myocardial infarction	54 (1.8%)	1,185 (0.9%)	25 (1.7%)	338 (1.4%)
Chronic kidney disease	279 (9.3%)	1,069 (0.8%)	152 (10.5%)	375 (1.6%)
COPD	169 (5.6%)	1,734 (1.3%)	118 (8.1%)	661 (2.8%)
Congestive heart failure	112 (3.7%)	1,233 (0.9%)	74 (5.1%)	454 (1.9%)
Diabetes mellitus	239 (7.9%)	3,259 (2.5%)	149 (10.3%)	781 (3.3%)
Essential hypertension	60 (2.0%)	1,497 (1.1%)	36 (2.5%)	364 (1.6%)
Fluid and electrolyte disorders	0 (0.0%)	1 (0.0%)	0 (0.0%)	0 (0.0%)
Lipid metabolism disorders	5 (0.1%)	235 (0.2%)	4 (0.3%)	50 (0.2%)
Lower respiratory disease	93 (3.1%)	931 (0.7%)	17 (1.2%)	1,581 (6.8%)
Pregnancy complications	21 (0.7%)	1,689 (1.3%)	58 (4.0%)	260 (1.1%)
**Involvement of medical specialty in the year prior to index event (Y0)** *no (%)*				
Cardiology	848 (28.1%)	17,000 (12.8%)	537 (37.0%)	4,965 (21.3%)
Cardiothoracic vascular surgery	125 (4.1%)	1,833 (1.4%)	88 (6.1%)	704 (3.0%)
Colorectal surgery	229 (7.6%)	3,194 (2.4%)	147 (10.1%)	1,331 (5.7%)
Endocrinology	312 (10.3%)	5,731 (4.3%)	200 (13.8%)	1,595 (6.8%)
Gastroenterology	599 (19.9%)	6,930 (5.2%)	320 (22.1%)	2,065 (8.8%)
General internal medicine	1,162 (38.5%)	16,102 (12.1%)	694 (47.8%)	4,595 (19.7%)
Medical oncology	182 (6.0%)	1,810 (1.4%)	86 (5.9%)	552 (2.4%)
Nephrology	330 (10.9%)	2,254 (1.7%)	185 (12.7%)	670 (2.9%)
Neurology	570 (18.9%)	11,975 (9.0%)	323 (22.2%)	3,290 (14.1%)
Ophthalmology	704 (23.3%)	16,607 (12.5%)	366 (25.2%)	3,473 (14.9%)
Radiation oncology	85 (2.8%)	1,287 (1.0%)	64 (4.4.%)	447 (1.9%)
Urology	423 (14.0%)	6,454 (4.9%)	278 (19.2%)	2,046 (8.8%)
**Healthcare costs**				
High-Cost status year 0^b^*; no (%)*	1,723 (57.0%)	9,106 (6.9%)	778 (53.6%)	3.165 (13.6%)
Total costs Y0; *median (IQR)*	€9,175 (€1,959-€18,029)	€277 (€0-€1,422)	€7,649 (€1,949-€17,435)	€1,012 (€183-€3,120)
Total costs Y1; *median (IQR)*	€16,424 (€11,468-€27,838)	€1,439 (€559-€3,609)	€20,686 (€13,024-€36,000)	€6,924 (€3,766-€11,804)
Total costs Y2; median* (IQR)*	€15,105 (€10,907-€26,067)	€480 (€0-€1,797)	€15,633 (€10,341-€28,710)	€853 (€135-€2,731)
Total costs Y3; *median (IQR)*	€14,395 (€9,744-€25,518)	€454 (€0-€1,740)	€15,282 (€10,000-€28,750)	€731 (€59-€2,518)

Characteristics for patients upon index event. Healthcare use, diagnoses and involved medical specialties were collected in a 365-day window prior to the index event (i.e. year 0)

*IQR* interquartile range, *CCI* Charlson comorbidity index, *ER* emergency room, *COPD* chronic obstructive pulmonary disease

^a^Persistent High-Cost = belonging to the top 10% of the cost distribution for three consecutive years

^b^High-Cost status year 0 = belonging to the top 10% of the cost distribution in year 0

**Fig. 2:**
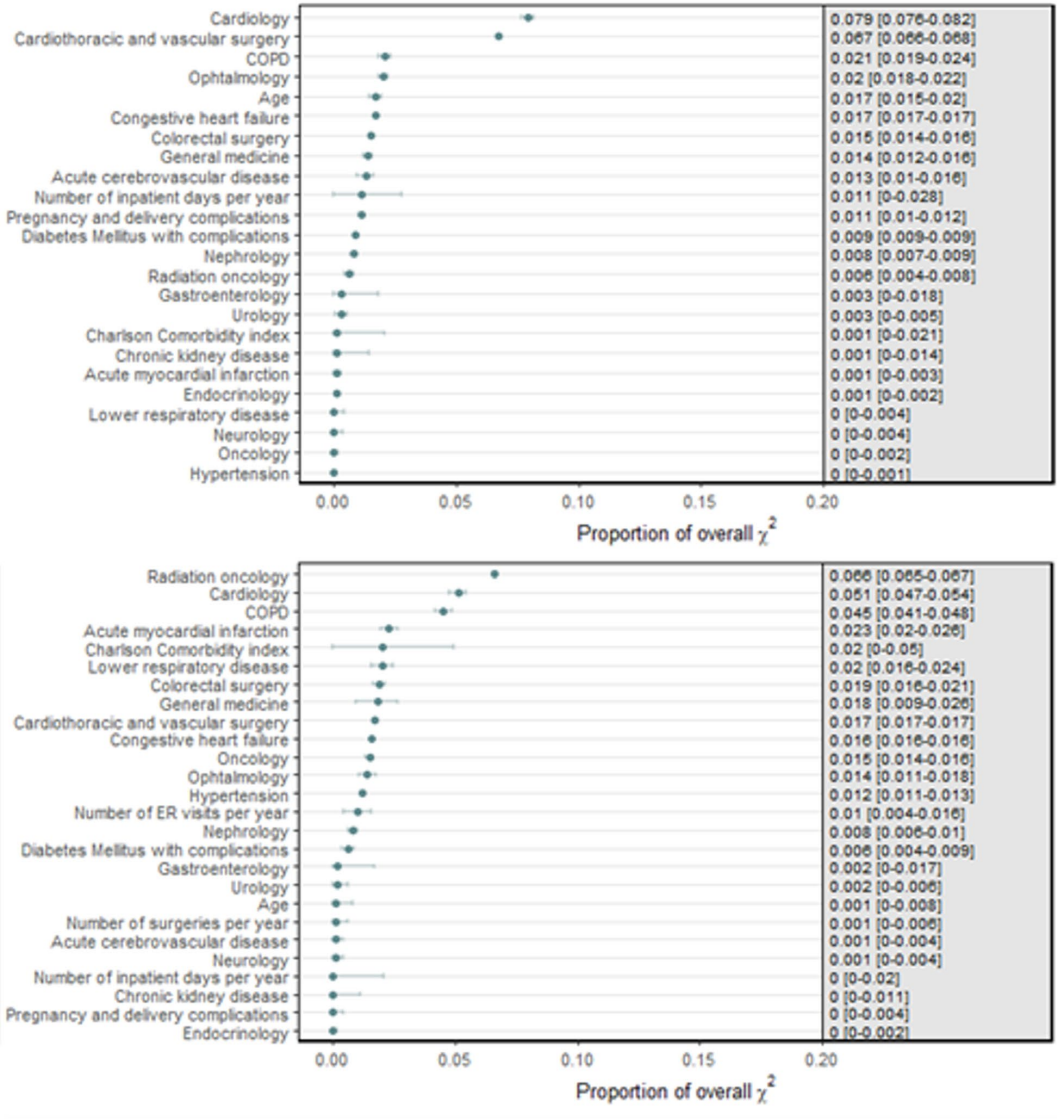
Individual predictor importance within the full hospital outpatient visit (top) and hospital admission (bottom) models. Individual predictors are ranked by their added predictive value to the model expressed through proportion of overall Wald χ2; the binary predicted outcome is Persistent High-Cost defined as belonging to the top 10% of the cost distribution for three consecutive years after the index event (i.e., outpatient visit (top panel) or hospital admission (bottom panel)); COPD = chronic obstructive pulmonary disease; ER = emergency room

Because lipid metabolism disorders and fluid and electrolyte disorders were registered in ≤ 0.5% of patients, these were dropped as predictors, resulting in 27 predictors in both full models (Table 1). The predictor with the strongest effect in both the outpatient visit—and hospital admission model was chronic kidney disease (OR 3.4 95%CI 2.6–4.5 and OR 2.1 95%CI 1.5–3.1, respectively) (Table 2). Full prediction models are provided in the appendices (Additional file 2). The predictive value added by individual predictors to each model (proportion of overall χ2) is depicted in Fig. 2.

**Table 2:** Results logistic multivariable regression analysis for the hospital outpatient visit—and hospital admission model

	**Hospital outpatient visit model**		**Hospital admission model**	
	**Full**	**Parsimonious**^**a**^	**Full**	**Parsimonious**^**a**^
	OR (95% CI)	OR (95% CI)	OR (95% CI)	OR (95% CI)
Sex *(female vs. male)*	0.93 (0.86—1.01)	-	0.91 (0.81—1.03)	-
Age at first visit *(per decade)*	0.95 (0.92—0.98)	0.98 (0.96—1.00)	0.90 (0.87—0.94)	-
CCI *(per point increase)*	1.35 (1.30—1.40)	-	1.28 (1.23—1.33)	1.32 (1.26—1.38)
**Healthcare use in the year prior**				
ER visits *(per visit)*	1.29 (1.18—1.40)	-	1.21 (1.11—1.31)	1.33 (1.23—1.44)
Surgeries *(per surgery)*	1.14 (1.10—1.17)	-	1.10 (1.06—1.15)	-
Inpatient days *(per day)*	1.01 (1.01—1.02)	1.02 (1.02—1.03)	1.01 (1.01—1.02)	-
**Diagnoses**				
Acute cerebrovascular disease	0.46 (0.34—0.62)	0.81 (0.60—1.08)	0.59 (0.40—0.86)	-
Acute myocardial infarction	0.69 (0.51—0.93)	-	0.47 (0.30—0.74)	0.48 (0.31—0.75)
Chronic kidney disease	3.40 (2.56—4.51)	4.37 (3.74—5.11)	2.15 (1.50—3.09)	2.80 (2.25—3.50)
COPD	2.09 (1.74—2.50)	2.86 (2.40—3.41)	1.80 (1.43—2.25)	1.77 (1.42—2.21)
Congestive heart failure	0.98 (0.77—1.23)	-	0.97 (0.72—1.30)	-
Diabetes Mellitus	1.09 (0.87—1.35)	-	1.19 (0.89—1.59)	-
Essential hypertension	0.77 (0.56—1.05)	-	0.80 (0.53—1.21)	-
Lower respiratory disease	2.31 (1.82—2.93)	-	1.97 (1.42—2.72)	2.16 (1.58—2.97)
Pregnancy complications	0.71 (0.46—1.10)	0.64 (0.41—0.99)	0.24 (0.15—0.40)	-
**Involvement of medical specialties**				
Cardiology	1.46 (1.33—1.62)	1.60 (1.46—1.75)	1.41 (1.23—1.62)	1.39 (1.23—1.57)
Cardiothoracic vascular surgery	1.01 (0.81—1.26)	-	0.96 (0.73—1.26)	-
Colorectal surgery	1.13 (0.96—1.33)	1.39 (1.19—1.63)	0.89 (0.72—1.10)	-
Endocrinology	0.83 (0.68—1.02)	-	0.82 (0.64—1.06)	-
Gastroenterology	2.61 (2.35—2.90)	3.05 (2.76—3.38)	1.88 (1.62—2.19)	2.00 (1.74—2.31)
General internal medicine	1.78 (1.58—20.1)	2.53 (2.32—2.76)	1.89 (1.61—2.22)	1.92 (1.69—2.19)
Nephrology	1.19 (0.90—1.57)	-	1.24 (0.87—1.76)	-
Neurology	1.63 (1.47—1.81)	-	1.32 (1.14—1.52)	-
Oncology	1.40 (1.27—1.55)	-	0.90 (0.67—1.21)	-
Ophthalmology	1.40 (1.27—1.55)	1.59 (1.45—1.75)	1.37 (1.19—1.58)	1.38 (1.21—1.58)
Radiation	0.78 (0.61—1.01)	1.55 (1.22—1.97)	1.01 (0.75—1.38)	-
Urology	1.57 (1.39—1.77)	-	1.51 (1.29—1.76)	-

Results on the binary outcome of belonging to the top 10% of the cost distribution for three consecutive years (i.e. Persistent High-Cost)

*OR* odds ratio, *CI* confidence interval, *CCI* Charlson comorbidity index, *ER* emergency room, *COPD* chronic obstructive pulmonary disease

^a^parsimonious = counterpart to full model with the lowest number of relevant predictors yet comparable performance

C-statistics in their respective development cohorts were 0.78 for the full outpatient visit model and 0.75 for the full hospital admission model. The parsimonious outpatient visit model consisted of 11 variables rather than the 27 from the full model and yielded a C-statistic of 0.74 in the development cohort. Similarly, the parsimonious hospital admission model consisted of 10 variables and yielded a C-statistic of 0.75 (Table 2). All C-statistics were corrected for optimism (i.e., internal validation) [28].

**Fig. 3:**
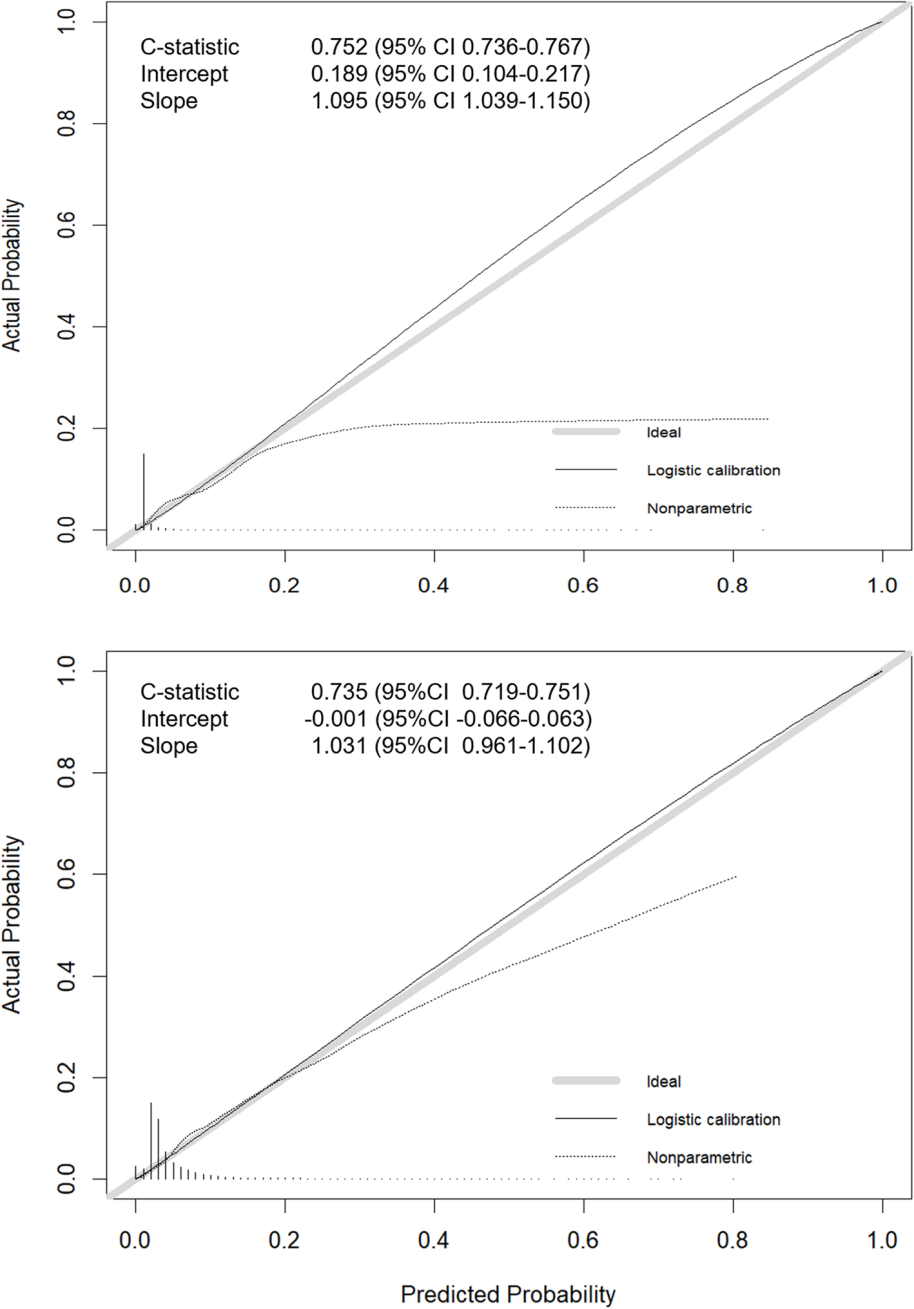
Calibration plots for the full hospital outpatient visit (top) and full hospital admission (bottom) models. Calibration represents the agreement between observed and predicted outcomes and was therefore assessed in the respective validation cohorts. Validation cohorts consisted of patients with their index event between July 2nd and December 31st, 2016. Continuous grey line represents perfect calibration while the continuous black line represents the model calibration curve. Intercept = calibration intercept (0 indicates perfect calibration); Slope = calibration slope (1 indicates perfect calibration)

The outpatient visit—and hospital admission temporal validation cohorts consisted of 84,009 and 20,768 patients, respectively. Percentage of patients becoming PHC was 1.5% (*n* = 1,270) and 5.0% (*n* = 1,039), respectively (Fig. 1). Temporal validation of the full models yielded a C-statistic of 0.75 for the outpatient visit model and 0.74 for the hospital admission model. Both were well-calibrated in their temporal validation cohorts: intercepts indicated good calibration for both models (0.19 and -0.01, respectively) [24, 27] (Fig. 3). Temporal validation of the parsimonious outpatient visit and hospital admission models yielded C-statistics of 0.70 and 0.73. Repeating all analyses with a High- Cost threshold of top 5% rather than top 10% showed similar results (Additional file 4). Sensitivity, specificity and positive predictive value for different risk cut-offs are reported in Additional file 5.

## Discussion

Persistent High-Cost (PHC), defined as belonging to the top 10% of the cost distribution for three consecutive years, can be predicted from routinely collected data upon a patient’s first outpatient visit to a medical specialist in the hospital and upon hospital admission. Both models, developed and validated in a large cohort, demonstrate good discrimination and calibration. Predictors are derived from administrative data based on international standards. As a result, both models are suitable for integration into Electronic Health Records (EHRs) as part of a hybrid case-finding strategy for High-Need High-Cost (HNHC) care management. By serving as a strong initial tier, these models can help optimize the assessment by healthcare professionals in the subsequent second tier. Ultimately, these models support a more targeted allocation of limited resources in care coordination for HNHC patients.

Baseline characteristics in the development cohorts were largely in line with other studies. First, the median age of patients who became PHC was only slightly higher compared to patients who did not, with the first quartile under fifty years of age in both groups (Additional file 3). This highlights that not only older but also younger adults can become PHC [21]. Second, nearly half (43%) of patients who became PHC did not belong to the top 10% of the cost distribution in their baseline year. Because our models, unlike previous studies, predict PHC irrespective of High-Cost status at baseline, there may be potential for early intervention or prevention [14]. Third, observed frequencies of diagnoses were generally lower than those in other studies. For example, only 1.1% (*n* = 1,557) and 1.6% (*n* = 400) had a diagnosis of hypertension in either development cohort, whereas Ng et al. found 25.7% (*n* = 39.184) and clinical prevalence is estimated to be even higher [14, 30]. This difference can be caused by the difference in data collection. We use claims data for diagnoses, which consist of ‘Diagnosis Treatment Combinations’ (DTCs) [20]. DTCs are established based on the main diagnosis, but often include more ‘minor’ diagnoses under the same umbrella. For example, if the DTC is renal insufficiency, this patient may have hypertension as well. This is noted in the clinical files but not registered separately in the claims data. Another explanation is that diagnoses are based on hospital care DTCs one year prior to the index event, possibly overlooking earlier diagnoses or those treated by general practitioners.

Comparing model performance with existing models is precarious because of differences in populations, definition and registration of predictors, and reported model performance measures [27]. Nonetheless, discrimination in the validation cohorts of both full models (C-statistics 0.75; 0.74) and their parsimonious counterparts (C-statistics 0.70; 0.73) is in line with C-statistics reported in other studies modelling PHC (range 0.68–0.82) [14–17]. Moreover, both models are well-calibrated in their validation cohorts, which is an important feature that is underreported for existing models [27].

This study has several limitations. First, we limited ourselves to those predictors that were included in the models identified in our systematic review and that were available in the claims database. We could therefore not include socioeconomic variables, mental health, or self-rated items as predictors while previous studies have underlined the importance of capturing medical and social complexities in case-finding for HNHC care management [2, 16]. Nevertheless, performance of the current models was adequate and this limitation may largely be mitigated by adopting a hybrid approach in which socioeconomic complexities can be addressed during individual assessments. Additionally, external validation and local updating could allow for extension with predictors that may be available in hospital data. Second, predictor effects were derived from administrative claims data, which could pose challenges when implementing models using different data sources. For example, we use registration of a hypertension DTC in our study as a predictor rather than a clinical diagnosis. Patients with hypertension as a ‘minor’ diagnosis instead of their primary disease (e.g. a patient with a stroke who also has hypertension), will not have a registration of a hypertension DTC. This limitation is underlined by the lower prevalence of diagnoses in our claims database than would be expected in clinical data. Third, our modelled outcome is healthcare cost rather than need. Because these do not necessary correlate completely (i.e. label bias), access to HNHC care management should not solely be based on model predictions [31]. Finally, a selection bias may be inferred from the exclusion of people who were not consecutively insured during the study period with the health insurer providing the data. This excluded group consists of people who died, switched health insurer or were incarcerated. As a result, patients with a high risk of becoming PHC (e.g., severely comorbid patients who died within two years) as well as patients with a low risk of becoming PHC (e.g., healthy young individuals who switched for budgetary reasons), were potentially excluded. Nevertheless, this limitation is less prominent when our models are part of a hybrid approach where limited life expectancy can be discussed and likely serves as an exclusion to care management [2, 4].

This study has several strengths. First, we provide two validated models, including their equations, predictor definitions, and operationalisation (Additional files 1 and 2). This information not only enables researchers to perform external validation of the models, it also allows for their integration into case-finding efforts HNHC care management. Second, by using five years of claims data we achieved a substantial sample size that represents all hospital-related cost, rather than being limited to cost from a single hospital. Third, adopting predictors from literature rather than using a data-driven approach to define and select our own, reduces the risk of overfitting [27]. Finally, limiting predictors to those that are based on international standards and available within the hospital setting, resulted in models that are suitable for implementation into EHRs. Risk-thresholds at which subsequent action is taken (e.g., inviting a patient for assessment), can be determined per hospital based on available resources and local care management goals, as a plausible threshold depends on the clinical context [32].

Our results have several implications for research and clinical practice. First, implementation of the models into care management should be preceded by external validation in settings distinct from those in which they were developed [33]. Second, it would be interesting to extend the models towards index events outside of the hospital setting such as primary care as HNHC care management programs are being developed in both settings. Third, exploring potential interactions between predictors and updating the model with sociodemographic predictors and other diagnoses such as mental health conditions may increase predictive performance further and could result in a more concise target population in a hybrid strategy. Finally, although refining models to improve predictive performance is important, future research should prioritize evaluating the added benefit of prediction models in hybrid case-finding. Considering that the ultimate goal is to impact patient care, employing decision-curve analysis could be a potential next step to assess clinical usefulness [27, 34, 35].

## Conclusions

In summary, we provide two validated models with good discrimination and calibration predicting PHC status upon patients’ first hospital outpatient visit and hospital admission. Because these models are based upon predictors that are often routinely collected in the hospital setting in an internationally universal way, they are suitable for integration into electronic health records. Future research should focus on implementation of prediction models into case-finding for HNHC care management as well as evaluation of clinical usefulness.

## Supplementary Information

Additional file 1. a. Definitions and operationalization of predictors. b. Operationalization of final predictors through CCSR codes.

Additional file 2. Regression formulas full hospital outpatient visit and hospital admission models.

Additional file 3. Distribution of age at baseline of patients who did and did not become Persistent High-Cost.

Additional file 4. Sensitivity analysis with High-Cost threshold of top 5%.

Additional file 5. Predictive probabilities of the hospital outpatient visit - and hospital admission model for different cut-off points.

## Data Availability

De-identified individual patient data that underlie the results reported in this paper and data dictionaries will be available following paper publication to investigators whose proposed use of the data has been approved by a learned intermediary. Proposals should be directed to the corresponding author (u.deruijter@erasmusmc.nl). Subject to third party permission, data requestors will need to sign a data access agreement to gain access. The study protocol, statistical plan, and analytic code (R script) are available from the corresponding author to anyone who wishes to access these documents immediately following publication without an end date.

## Abbreviations

COVID-19: Coronavirus disease 2019
CCSR: Clinical classifications software refined
HER: Electronic health records
DTC: Diagnosis treatment combinations
DRG: Diagnosis related groups
EHR: Electronic health records
ER: Emergency room
HNHC: High-need high-cost
OR: Odds ratio
PHC: Persistent high-cost
TRIPOD: Transparent reporting of a multivariable prediction model for individual prognosis or diagnosis
