# Supplementary material for: Identifying persistent high-cost patients in the hospital for care management: development and validation of prediction models
Source: BMC Health Serv Res. 2024 Nov 26;24:1469. doi: 10.1186/s12913-024-11936-7 (PMC11590622; doi:10.1186/s12913-024-11936-7)
Supplement: Supplementary file 1 — Additional file 1. a. Definitions and operationalization of predictors. b. Operationalization of final predictors through CCSR codes. [file 12913_2024_11936_MOESM1_ESM.zip › Additional file 1a.docx]

**Additional file 1 - Definitions and operationalization of predictors**

All predictors from the studies by Ng et al. (Singapore), Robst (United States of America) and Rosella et al. (Canada) were considered for model building.^1-4^ All predictors and their definitions are listed below. Operationalization of the final predictors in our models through CCS codes are listed in the accompanying Excel file.

**Additional file Table 1a** - Definitions of predictors included in the Persistent High Utilizer (PHU) model predicting belonging to the top-10% of total annual hospital cost for two consecutive years, after having been HU already for a year by Ng et al.^1^

| **Predictor** | **Definition** |
| --- | --- |
| Age | Age at first visit |
| Charlson Comorbidity Score | Maximum Charlson Comorbidity Score of corresponding patient, considering any given visit |
| Polypharmacy score | Maximum total number of unique drugs prescribed during any given visit |
|  |  |
| **MEDICAL SPECIALTY INVOLVEMENT** | |
| Cardiology | Binary indicator of whether the visit was at the department of cardiology |
| Cardiothoracic & vascular surgery | Binary indicator of whether the visit was at the department of cardiothoracic & vascular surgery |
| Colorectal surgery | Binary indicator of whether the visit was at the department of colorectal surgery |
| Emergency medicine | Binary indicator of whether the visit was at the department of emergency medicine |
| Endocrinology | Binary indicator of whether the visit was at the department of endocrinology |
| Gastroenterology | Binary indicator of whether the visit was at the department of gastroenterology |
| General medicine | Binary indicator of whether the visit was at the department of general medicine |
| Medical oncology | Binary indicator of whether the visit was at the department of medical oncology |
| Nephrology | Binary indicator of whether the visit was at the department of nephrology |
| Neurology | Binary indicator of whether the visit was at the department of neurology |
| Ophthalmology | Binary indicator of whether the visit was at the department of ophthalmology |
| Radiation oncology | Binary indicator of whether the visit was at the department of radiation oncology |
| Urology | Binary indicator of whether the visit was at the department of urology |
|  |  |
| **DIAGNOSES*** | |
| Acute myocardial infarction (chronic) | Binary indicator of whether the visit has a primary or secondary diagnosis in CCS grouping "Acute myocardial infarction" |
| Acute cerebrovascular disease (chronic) | Binary indicator of whether the visit has a primary or secondary diagnosis in CCS grouping "Acute cerebrovascular disease" |
| Disorders of lipid metabolism (chronic) | Binary indicator of whether the visit has a primary or secondary diagnosis in CCS grouping "Disorders of lipid metabolism" |
| Diabetes mellitus with complication (chronic) | Binary indicator of whether the visit has a primary or secondary diagnosis in CCS grouping "Diabetes mellitus with complications" |
| Chronic kidney disease (chronic) | Binary indicator of whether the visit has a primary or secondary diagnosis in CCS grouping "Chronic renal failure" |
| COPD and bronchiectasis (chronic) | Binary indicator of whether the visit has a primary or secondary diagnosis in CCS grouping "Chronic obstructive pulmonary disease and bronchiectasis" |
| Congestive heart failure nonhypertensive (chronic) | Binary indicator of whether the visit has a primary or secondary diagnosis in CCS grouping "Congestive heart failure; nonhypertensive" |
| Essential hypertension (chronic) | Binary indicator of whether the visit has a primary or secondary diagnosis in CCS grouping "Essential hypertension" |
| Fluid and electrolyte disorders (chronic) | Binary indicator of whether the visit has a primary or secondary diagnosis in CCS grouping "Fluid and electrolyte disorders" |
| Other lower respiratory disease (acute) | Binary indicator of whether the visit has a primary or secondary diagnosis in CCS grouping "Other lower respiratory disease" |
| Other pregnancy and delivery (acute) | Binary indicator of whether the visit has a primary or secondary diagnosis in CCS grouping "Other pregnancy and delivery including normal" |
|  |  |
| **PRIOR HEALTHCARE USE** | |
| Number of surgeries | Total number of days with a surgery of patient (max 365) |
| Length of stay | Cumulative length-of-stay of inpatient admissions (0 for outpatient visits) |
| Emergency attendances | Total number of visits to emergency department (ED) of patient |
| Average inpatient expenditure | Average inflation-adjusted hospital charges of inpatient services |
| Average outpatient expenditure | Average inflation-adjusted hospital charges of outpatient services |
| Consumables/Appliances/Implants expenditure | Inflation-adjusted hospital charges of consumables/appliances/implants fee of the visit |
| Dialysis expenditure | Inflation-adjusted hospital charges of dialysis of patient |
| Doctor fee expenditure | Inflation-adjusted hospital charges of doctor fee of patient |
| Doctor fee expenditure (%) | Relative inflation-adjusted hospital charges of doctor fee of patient |
| Inpatient expenditure | Inflation-adjusted hospital charges of ward facility of the visit |
| Laboratory expenditure | Inflation-adjusted hospital charges of lab investigations of the visit |
| Laboratory expenditure (%) | Relative inflation-adjusted hospital charges of lab investigations of the visit |
| Other services expenditure | Inflation-adjusted hospital charges of other services of the visit |
| Outpatient expenditure | Inflation-adjusted hospital charges of outpatient services of the visit |
| Overall expenditure | Inflation-adjusted hospital charges of patient |
| Prescription expenditure | Inflation-adjusted hospital charges of prescriptions of patient |
| Prescription expenditure (%) | Relative inflation-adjusted hospital charges of prescriptions of patient |
| Procedure expenditure | Inflation-adjusted hospital charges of procedure fee of patient |
| Procedure expenditure (%) | Relative inflation-adjusted hospital charges of procedure fee of patient |
| Specialized investigations expenditure | Inflation-adjusted hospital charges of specialized investigations of patient |
| Surgical procedure expenditure | Inflation-adjusted hospital charges of surgical procedures of patient |
| Surgical procedure expenditure (%) | Relative inflation-adjusted hospital charges of surgical procedures of patient |
| Therapy expenditure (%) | Relative inflation-adjusted hospital charges of therapies of the visit |
| X-ray expenditure (%) | Relative inflation-adjusted hospital charges of radiologic exams of the visit |

**As defined by Clinical Classification Software (CCS) and Chronic Condition Indicator (CCI) by Agency for Healthcare Research and Quality*

**Additional file Table 1b** - Definitions of predictors included in the High Resource User Population Risk Tool (HRUPoRT), a population-based risk prediction tool for transition to the top 5% of health resource users over a 5-year period by Rosella et al.^2,4^

| **Predictor** | **Levels** | **Levels** | **Definition** |
| --- | --- | --- | --- |
| Sex | 2 | M vs. F | Male vs. female |
| Age at first visit | 7 | <30; 30-39; 40-49; 50-59; 60-69; 70-79; ≥80 | Age in years at baseline |
| Ethnicity | 2 | White | Are you white? |
|  |  | Non-white | Are you white? |
| Immigrant status | 3 | Canadian-born | Were you born a Canadian citizen? |
|  |  | Immigrant < 10 years | Were you born a Canadian citizen?  In what year did you first come to Canada to live? |
|  |  | Immigrant ≥ 10 years | Were you born a Canadian citizen? No  In what year did you first come to Canada to live? |
| Household income | 4 | First quartile; second quartile; third quartile; fourth quartile | The adjusted ratio of their total household income to the low-income cut-off corresponding to their household community size |
| Food security* | 2 | Food secure vs. insecure | Household food security index |
| Chronic conditions | 2 | Yes vs. no |  |
| General health | 3 | Excellent, very good and good | In general, would you say your health is excellent, very good, good, fair, or poor? |
|  |  | Fair | In general, would you say your health is excellent, very good, good, fair, or poor? |
|  |  | Poor | In general, would you say your health is excellent, very good, good, fair, or poor? |
| BMI^*^ | 6 | Underweight | <18.5 kg/m2 |
|  |  | Normal weight | 18.5-24.9 kg/m2 |
|  |  | Overweight | 25.0-29.9 kg/m2 |
|  |  | Moderately obese | 30.0-34.9 kg/m2 |
|  |  | Very obese | 35.0-39.9 kg/m2 |
|  |  | Severely obese | ≥ 40.0 kg/m2 |
| Smoking status^†^ | 5 | Heavy smoker | Current smoker [≥1 pack (25 cigarettes)/d] |
|  |  | Light smoker | Current smoker [< 1 pack (25 cigarettes)/d] |
|  |  | Former heavy smoker | Former smoker [≥ 1 pack (25 cigarettes)/d] |
|  |  | Former light smoker | Former smoker [< 1 pack (25 cigarettes)/d] |
|  |  | Nonsmoker | Never smoker or former occasional smoker with <100 lifetime cigarettes |
| Physical activity^‡^ | 4 | First quartile | Bottom 25% physically active |
|  |  | Second quartile | Bottom 26%–50% physically active |
|  |  | Third quartile | Bottom 51%–75% physically active |
|  |  | Fourth quartile | Top 25% physically active |
| Alcohol consumption^§^ | 4 | Heavy drinker | ≥21 (men) drinks per week in the past 12 months or ≥14 (women) drinks in the  previous week, or binging behavior on a weekly basis (≥5 drinks on any  occasion) |
|  |  | Moderate drinker | 4–21 (men) or 3–14 (women) drinks in the  previous week |
|  |  | Light drinker | 1–3 (men) or 1–2 (women) drinks in the previous week |
|  |  | Nondrinker | No alcohol consumption in the last 12 months |
| Education | 3 | Less than secondary | What is the highest degree, certificate, or diploma you have obtained? |
|  |  | Secondary graduate | What is the highest degree, certificate, or diploma you have obtained? |
|  |  | More than secondary | What is the highest degree, certificate, or diploma you have obtained? |
| Marital status | 2 | Married/common law vs. other | What is your marital status? Are you married, living common-law, widowed, separated, divorced, single, or never married |
| Perceived mental health | 2 | Good vs. poor | In general, would you say your mental health is excellent, very good, good, fair, or poor? |
| Consulted mental health professional | 2 | Yes vs. no | n the past 12 months have you seen or talked to a health professional about your emotional or mental health? |
| Has a regular doctor | 2 | Yes vs. no | Do you have a regular medical doctor? |
| Life satisfaction | 5 | Very satisfied | How do you feel about your life as a whole right now – very satisfied, satisfied, neither satisfied nor dissatisfied, dissatisfied, very dissatisfied? |
|  |  | Satisfied | How do you feel about your life as a whole right now – very satisfied, satisfied, neither satisfied nor dissatisfied, dissatisfied, very dissatisfied? |
|  |  | Neither | How do you feel about your life as a whole right now – very satisfied, satisfied, neither satisfied nor dissatisfied, dissatisfied, very dissatisfied? |
|  |  | Dissatisfied | How do you feel about your life as a whole right now – very satisfied, satisfied, neither satisfied nor dissatisfied, dissatisfied, very dissatisfied? |
|  |  | Very dissatisfied | How do you feel about your life as a whole right now – very satisfied, satisfied, neither satisfied nor dissatisfied, dissatisfied, very dissatisfied? |
| Perceived life stress | 2 | High vs. low | Thinking about the amount of stress in your life, would you say that the days are not at all stressful, not very stressful, a bit stressful, quite a bit stressful, or extremely stressful? |
| Diet score^\|\|^ | 4 | Lowest | <1.0 |
|  |  | Low | 1.0-1.9 |
|  |  | High | 2.0-2.9 |
|  |  | Highest | ≥3.0 |

** Calculated using self-reported height and weight.*

*† On the basis of self-reported smoking behavior*

*‡ On the basis of average daily metabolic equivalent of task expenditure (kcal/kg/d) for participation in an aggregate list of leisure time physical activities in the past 3 months.*

*§ On the basis of self-reported drinking behavior*

*|| Derived from self-reported daily consumption of carrots, potatoes, fruit juice and total fruits and vegetables.*

**Additional file Table 1c** - Definitions of predictors included in the models to predict Persistent High-Cost cases in Florida Medicaid by Robst* ^3^

| **Predictor** | **Levels** | **Levels** | **Definition** |
| --- | --- | --- | --- |
| Age at first visit | C |  |  |
| Race | 5 | White |  |
|  |  | Asian |  |
|  |  | Black |  |
|  |  | Hispanic |  |
|  |  | Other | The Florida Medicaid race variable has a number of individuals with unknown race who are likely to be Hispanic. However, instead of simply assuming they are Hispanic, the race variable was coded as ‘‘other.’’ |
| Eligibility | 2 | Supplemental Security Income | NA |
|  |  | Long-term care | NA |
| Behavioral health utilization | 3 | Inpatient | NA |
|  |  | Outpatient | NA |
|  |  | Pharmacy | NA |
| Physical health utilization | 7 | Inpatient | NA |
|  |  | Outpatient | NA |
|  |  | Other | NA |
|  |  | Pharmacy | NA |
|  |  | Nursing home | NA |
|  |  | Hospice | NA |
|  |  | Intermediate Case Facility - Mental Retardation | NA |
| Diagnoses | 18 | Infectious diseases | ICD-9 001–139 |
|  |  | Neoplasms | ICD-9 140–239 |
|  |  | Endocrine diseases | ICD-9 240–279 |
|  |  | Diseases of blood | ICD-9 280–289 |
|  |  | Mental disorders | ICD-9 290–319 |
|  |  | Nervous system | ICD-9 320–359 |
|  |  | Sense organs | ICD-9 360–389 |
|  |  | Circulatory system | ICD-9 390–459 |
|  |  | Respiratory system | ICD-9 460–519 |
|  |  | Digestive system | ICD-9 520–579 |
|  |  | Genitourinary system | ICD-9 580–629 |
|  |  | Pregnancy complication | ICD-9 630–679 |
|  |  | Skin diseases | ICD-9 680–709 |
|  |  | Musculoskeletal | ICD-9 710–739 |
|  |  | Congenital anomalies | ICD-9 740–759 |
|  |  | Perinatal conditions | ICD-9 760–779 |
|  |  | Ill-defined conditions | ICD-9 780–799 |
|  |  | Injury/poisoning | ICD-9 800–999 |

** ICD-9 = International Classification of Disease, 9^th^ edition*

**References**

1. Ng SHX, Rahman N, Ang IYH, et al. Characterising and predicting persistent high-cost utilisers in healthcare: A retrospective cohort study in singapore. *BMJ open*. 2020;10(1):e031622.

2. Rosella LC, Kornas K, Sarkar J, Fransoo R. External validation of a population-based prediction model for high healthcare resource use in adults. . 2020;8(4):537.

3. Robst J. Developing models to predict persistent high-cost cases in florida medicaid. *Population health management*. 2015;18(6):467-476.

4. Rosella LC, Kornas K, Yao Z, et al. Predicting high health care resource utilization in a single-payer public health care system: Development and validation of the high resource user population risk tool. *Med Care*. 2018;56(10):e61.
