# Supplementary material for: Identifying persistent high-cost patients in the hospital for care management: development and validation of prediction models
Source: BMC Health Serv Res. 2024 Nov 26;24:1469. doi: 10.1186/s12913-024-11936-7 (PMC11590622; doi:10.1186/s12913-024-11936-7)
Supplement: Supplementary file 2 — Additional file 2. Regression formulas full hospital outpatient visit and hospital admission models. [file 12913_2024_11936_MOESM2_ESM.docx]

**Additional file 2 - Regression formulas full hospital outpatient visit and full hospital admission models**

|  | **Hospital outpatient visit model** | | | | **Hospital admission model** | | | |
| --- | --- | --- | --- | --- | --- | --- | --- | --- |
|  | **Full** | | **Parsimonious** | | **Full** | | **Parsimonious** | |
|  | **β** | ***p*** | **β** | ***p*** | **β** | ***p*** | **β** | ***p*** |
| Constant | -8.769 | <0.0001 | -9.915 | <0.0001 | -4.634 | <0.0001 | -7.242 | <0.0001 |
| Sex *(female)* | -0.073 | 0.07 | - | - | -0.090 | 0.13 | - | - |
| Age at first visit; *per decade* | -0.048 | <0.0001 | -0.021 | 0.11 | -0.103 | <0.0001 | - | - |
| Charlson Comorbidity Index; *per point* | 0.301 | <0.0001 | - | - | 0.252 | <0.0001 | 0.277 | <0.0001 |
| **PRIOR HEALTHCARE USE** | | | | | | | | |
| Surgeries; *per surgery* | 0.130 | <0.0001 | - | - | 0.098 | <0.0001 | - | - |
| ER visits; *per visit* | 0.252 | <0.0001 | - | - | 0.188 | <0.0001 | 0.286 | <0.0001 |
| Inpatient days; *per day* | 0.012 | <0.0001 | 0.022 | <0.0001 | 0.012 | <0.0001 | - | - |
| **DIAGNOSES** | | | | | | | | |
| Acute cerebrovascular disease | -0.771 | <0.0001 | -0.216 | 0.14 | -0.536 | 0.01 | - | - |
| Acute myocardial infarction | -0.375 | 0.02 | - | - | -0.747 | <0.0001 | -0.728 | <0.0001 |
| Chronic kidney disease | 1.224 | <0.0001 | 1.476 | <0.0001 | 0.766 | <0.0001 | 1.031 | <0.0001 |
| COPD | 0.736 | <0.0001 | 1.051 | <0.0001 | 0.585 | <0.0001 | 0.570 | <0.0001 |
| Congestive heart failure | -0.025 | 0.83 | - | - | -0.032 | 0.83 | - | - |
| Diabetes Mellitus | 0.085 | 0.45 | - | - | 0.174 | 0.24 | - | - |
| Essential hypertension | -0.261 | 0.10 | - | - | -0.223 | 0.29 | - | - |
| Lower respiratory disease | 0.836 | <0.0001 | - | - | 0.677 | <0.0001 | 0.772 | <0.0001 |
| Pregnancy complications | -0.343 | 0.13 | -0.451 | 0.04 | -1.407 | <0.0001 | - | - |
| **INVOLVEMENT OF MEDICAL SPECIALTY** | | | | | | | | |
| Cardiology | 0.382 | <0.0001 | 0.469 | <0.0001 | 0.345 | <0.0001 | 0.331 | <0.0001 |
| Cardiothoracic surgery | 0.013 | 0.91 | - | - | -0.041 | 0.77 | - | - |
| Colorectal surgery | 0.122 | 0.15 | 0.331 | <0.0001 | -0.112 | 0.29 | - | - |
| Endocrinology | -0.183 | 0.07 | - | - | -0.196 | 0.13 | - | - |
| General medicine | 0.577 | <0.0001 | 0.928 | <0.0001 | 0.635 | <0.0001 | 0.653 | <0.0001 |
| Gastroenterology | 0.960 | <0.0001 | 1.117 | <0.0001 | 0.634 | <0.0001 | 0.694 | <0.0001 |
| Nephrology | 0.175 | 0.22 | - | - | 0.216 | 0.23 | - | - |
| Neurology | 0.488 | <0.0001 | - | - | 0.275 | <0.0001 | - | - |
| Oncology | 0.336 | <0.0001 | - | - | -0.105 | 0.49 | - | - |
| Ophthalmology | 0.338 | <0.0001 | 0.466 | <0.0001 | 0.314 | <0.0001 | 0.323 | <0.0001 |
| Radiation | -0.244 | 0.06 | 0.439 | <0.0001 | 0.014 | 0.93 | - | - |
| Urology | 0.451 | <0.0001 | - | - | 0.410 | <0.001 | - | - |

**Additional file Table 2** – Regression coefficients for logistic multivariable regression analysis on the binary outcome of belonging to the top 10% of the cost distribution for three consecutive years for both the hospital outpatient visit - and hospital admission model.

*β = regression coefficient; ER = emergency room.*
