# Supplementary material for: Identifying persistent high-cost patients in the hospital for care management: development and validation of prediction models
Source: BMC Health Serv Res. 2024 Nov 26;24:1469. doi: 10.1186/s12913-024-11936-7 (PMC11590622; doi:10.1186/s12913-024-11936-7)
Supplement: Supplementary file 3 — Additional file 3. Distribution of age at baseline of patients who did and did not become Persistent High-Cost. [file 12913_2024_11936_MOESM3_ESM.docx]

**Additional file 3** Distribution of age at baseline of patients who did and did not become Persistent High-Cost


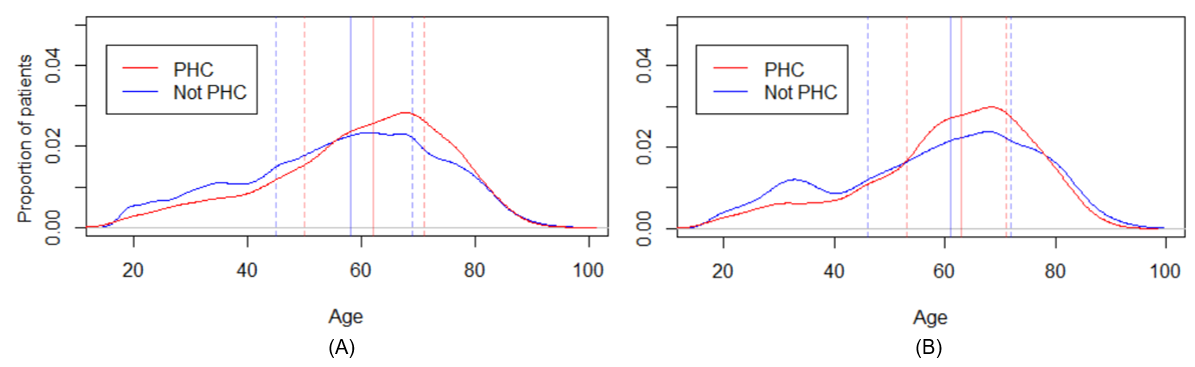


*Panel A - distribution of age in the hospital outpatient visit development cohort (n = 135.558). Panel B - distribution of age in the hospital admission development cohort (n = 24.805). Continuous vertical lines represent the median age of that specific cohort while the dashed vertical lines represent the respective first and third quartile; PHC = persistent high-cost, defined as belonging to the top 10% of the cost distribution for three consecutive years.*
