# Supplementary material for: Identifying persistent high-cost patients in the hospital for care management: development and validation of prediction models
Source: BMC Health Serv Res. 2024 Nov 26;24:1469. doi: 10.1186/s12913-024-11936-7 (PMC11590622; doi:10.1186/s12913-024-11936-7)
Supplement: Supplementary file 4 — Additional file 4. Sensitivity analysis with High-Cost threshold of top 5%. [file 12913_2024_11936_MOESM4_ESM.docx]

**Additional file 4 - Sensitivity analysis with High-Cost threshold of top-5%**

**Additional file Table 3a** – Characteristics of patients with and without Persistent High-Cost status in their baseline year in the outpatient visit development cohort using a High-Cost threshold of top-5%.

|  | **Outpatient population**  **(*n* = 135.558)** | |
| --- | --- | --- |
|  | **Persistent High-Cost**  **using top 5% threshold**  **(*n* = 1.300)** | **Others**  **using top 5% threshold**  **(*n* = 134.258)** |
| Sex; *no of females (%)* | 711 (54.7%) | 84.089 (62.6%) |
| Age in years; *median (IQR)* | 62 (50-71) | 58 (45-69) |
| CCI; *median (IQR)* | 1 (0-2) | 0 (0-0) |
| No. of ER visits; *median (IQR)* | 0 (0-0) | 0 (0-0) |
| No. of surgeries; *median (IQR)* | 0 (0-1) | 0 (0-0) |
| No. of inpatient days; *median (IQR)* | 0 (0-4) | 0 (0-0) |
| **INVOLVEMENT OF MEDICAL SPECIALTY** *no (%)* | | |
| Cardiology | 374 (28.8%) | 17.474 (13%) |
| Cardiothoracic & vascular surgery | 51 (3.9%) | 1.907 (1.4%) |
| Colorectal surgery | 100 (7.7%) | 3.323 (2.5%) |
| Endocrinology | 135 (10.4%) | 5.908 (4.4%) |
| Gastroenterology | 268 (20.6%) | 7.261 (5.4%) |
| General medicine | 607 (46.7%) | 16.657 (12.4%) |
| Medical oncology | 104 (8.0%) | 1.888 (1.4%) |
| Nephrology | 223 (17.2%) | 2.361 (1.8%) |
| Neurology | 259 (19.9%) | 12.286 (9.1%) |
| Ophthalmology | 309 (23.8%) | 17.002 (12.7%) |
| Radiation oncology | 37 (2.8%) | 1.335 (1.0%) |
| Urology | 191 (14.7%) | 6.686 (5.0%) |
| **DIAGNOSES** *no (%)* | | |
| Acute cerebrovascular disease | 21 (1.6%) | 1.452 (1.1%) |
| Acute myocardial infarction | 21 (1.6%) | 1.218 (0.9%) |
| Chronic kidney disease | 210 (16.2%) | 1.138 (0.8%) |
| COPD | 81 (6.2%) | 1.822 (1.4%) |
| Congestive heart failure | 56 (4.3%) | 1.289 (1.0%) |
| Diabetes mellitus | 109 (8.4%) | 3.389 (2.5%) |
| *Disorders of lipid metabolism* | *2 (0.2%)* | *238 (0.2%)* |
| Essential hypertension | 28 (2.2%) | 1.529 (1.1%) |
| *Fluid and electrolyte disorders* | *0 (0.0%)* | *1 (0.0%)* |
| Lower respiratory disease | 39 (3.0%) | 985 (0.7%) |
| Pregnancy complications | 11 (0.8%) | 1.699 (1.3%) |
| **EXPENDITURE** | | |
| High-Cost status Year 0^†^; *no (%)* | 722 (55.6%) | 5.087 (3.8%) |
| Total expenditure Y0; *median (IQR)* | €14.042 (€2.938-€27.297) | €1.987 (€0-€1.484) |
| Total expenditure Y1; *median (IQR)* | €24.918 (€16.627-€45.262) | €1.471 (€566-€3.759) |
| Total expenditure Y2; median *(IQR)* | €22.943 (€15.854-€48.893) | €497 (€0-€1.889) |
| Total expenditure Y3; *median (IQR)* | €22.998 (€15.479-€47.806) | €469 (€0-€1.832) |

*IQR = interquartile range; CCI = Charlson comorbidity index; ER = emergency room; COPD = chronic obstructive pulmonary disease; italics indicate predictors that were discarded because of an occurrence of ≤0.5% at baseline*

**Additional file Table 3b** – Characteristics of patients with and without Persistent High-Cost status in their baseline year in the hospital admission development cohort using a High-Cost threshold of top-5%.

|  | **Hospital admission population**  **(*n* = 24.805)** | |
| --- | --- | --- |
|  | **Persistent High-Cost**  **using top 5% threshold**  **(*n* = 661)** | **Others**  **using top 5% threshold**  **(*n* = 24.144)** |
| Sex; *no of females (%)* | 336 (50.8%) | 14.572 (60.4%) |
| Age in years; *median (IQR)* | 62 (51-70) | 61 (47-72) |
| CCI; *median (IQR)* | 0 (1-2) | 0 (0-0) |
| No. of ER visits; *median (IQR)* | 0 (0-0) | 0 (0-0) |
| No. of surgeries; *median (IQR)* | 0 (0-1) | 0 (0-0) |
| No. of inpatient days; *median (IQR)* | 0 (0-9) | 0 (0-0) |
| **INVOLVEMENT OF MEDICAL SPECIALTY** *no (%)* | | |
| Cardiology | 250 (37.8%) | 5.252 (21.8%) |
| Cardiothoracic & vascular surgery | 42 (6.4%) | 750 (3.1%) |
| Colorectal surgery | 70 (10.6%) | 1.408 (5.8%) |
| Endocrinology | 86 (13.0%) | 1.709 (7.1%) |
| Gastroenterology | 162 (24.5%) | 2.223 (9.2%) |
| General medicine | 363 (54.9%) | 4.926 (20.4%) |
| Medical oncology | 49 (7.4%) | 589 (2.4%) |
| Nephrology | 122 (18.5%) | 733 (3.0%) |
| Neurology | 140 (21.1%) | 3.473 (14.4%) |
| Ophthalmology | 170 (25.7%) | 3.669 (15.2%) |
| Radiation oncology | 22 (3.3.%) | 489 (2.0%) |
| Urology | 133 (20.1%) | 2.191 (9.1%) |
| **DIAGNOSES** *no (%)* | | |
| Acute cerebrovascular disease | 16 (2.4%) | 442 (1.8%) |
| Acute myocardial infarction | 13 (2.0%) | 350 (1.4%) |
| Chronic kidney disease | 112 (16.9%) | 415 (1.7%) |
| COPD | 53 (8.0%) | 726 (3.0%) |
| Congestive heart failure | 40 (6.1%) | 488 (2.0%) |
| Diabetes mellitus | 75 (11.3%) | 855 (3.5%) |
| *Disorders of lipid metabolism* | *1 (0.0%)* | *53 (0.2%)* |
| Essential hypertension | 17 (2.6%) | 383 (1.6%) |
| *Fluid and electrolyte disorders* | *0 (0.0%)* | *0 (0.0%)* |
| Lower respiratory disease | 33 (5.0%) | 285 (1.2%) |
| Pregnancy complications | 6 (0.9%) | 1.592 (6.6%) |
| **EXPENDITURE** | | |
| High-Cost status Year 0^†^; *no (%)* | 322 (48.7%) | 1.908 (7.9%) |
| Total expenditure Y0; *median (IQR)* | €10.902 (€2.658-€25.218) | €1.063 (€193-€3.340) |
| Total expenditure Y1; *median (IQR)* | €30.680 (€19.328-€52.258) | €7.210 (€3.829-€12.236) |
| Total expenditure Y2; median *(IQR)* | €24.949 (€16.667-€52.258) | €933 (€147-€3.089) |
| Total expenditure Y3; *median (IQR)* | €23.363 (€15.907-€51.435) | €795 (€89-€2.885) |

*IQR = interquartile range; CCI = Charlson comorbidity index; ER = emergency room; COPD = chronic obstructive pulmonary disease; italics indicate predictors that were discarded because of an occurrence of ≤0.5% at baseline*

**Additional file Table 3c** – Results logistic multivariable regression analysis on the binary outcome of belonging to the top 5% of the cost distribution for three consecutive years for both the hospital outpatient visit - and hospital admission model.^*^

|  | **Full hospital outpatient visit model using top 5% High-Cost threshold** | **Full hospital admission model using top 5% High-Cost threshold** |
| --- | --- | --- |
|  | OR (95% CI) | OR (95% CI) |
| Sex *(female vs. male)* | 0.88 (0.78-0.99) | 0.89 (0.75-1.05) |
| Age at first visit *(per decade)* | 0.92 (0.89-0.96) | 0.85 (0.80-0.90) |
| CCI *(per point increase)* | 1.36 (1.30-1.43) | 1.31 (1.23-1.41) |
| **PRIOR HEALTHCARE USE** | | |
| Number of surgeries | 1.12 (1.08-1.17) | 1.09 (1.03-1.15) |
| Number of ER attendances | 1.20 (1.07-1.34) | 1.17 (1.05-1.31) |
| Number of inpatient days | 1.01 (1.01-1.02) | 1.01 (1.01-1.02) |
| **DIAGNOSES** | | |
| Acute cerebrovascular disease | 0.34 (0.21-0.55) | 0.61 (0.35-1.07) |
| Acute myocardial infarction | 0.62 (0.38-1.00) | 0.58 (0.32-1.08) |
| Chronic kidney disease | 0.63 (0.44-0.91) | 3.72 (2.36-5.85) |
| COPD | 0.22 (0.17-0.29) | 1.66 (1.20-2.29) |
| Congestive heart failure | 0.92 (0.66-1.28) | 1.00 (0.67-1.49) |
| Diabetes Mellitus | 1.14 (0.83-1.58) | 1.54 (1.02-2.33) |
| Essential hypertension | 0.84 (0.54-1.30) | 0.87 (0.49-1.53) |
| Lower respiratory disease | 1.90 (1.33-2.71) | 2.22 (1.47-3.36) |
| Pregnancy complications | 0.89 (0.48-1.63) | 0.19 (0.08-0.43) |
| **INVOLVED MEDICAL SPECIALTIES** | | |
| Cardiology | 1.33 (1.15-1.55) | 1.29 (1.06-1.57) |
| Cardiothoracic surgery | 0.83 (0.60-1.17) | 0.90 (0.61-1.32) |
| Colorectal surgery | 1.05 (0.82-1.35) | 0.91 (0.68-1.22) |
| Endocrinology | 0.69 (0.51-0.93) | 0.54 (0.37-0.78) |
| Gastroenterology | 2.49 (2.13-2.91) | 2.06 (1.68-2.53) |
| General medicine | 2.16 (1.81-2.57) | 2.25 (1.79-2.82) |
| Nephrology | 1.06 (0.73-1.53) | 1.07 (0.68-1.69) |
| Neurology | 1.70 (1.46-1.98) | 1.15 (0.93-1.42) |
| Oncology | 1.64 (1.25-2.15) | 1.09 (0.74-1.61) |
| Ophthalmology | 1.37 (1.18-1.59) | 1.35 (1.10-1.66) |
| Radiation | 0.63 (0.44-0.92) | 0.56 (0.35-0.92) |
| Urology | 1.45 (1.22-1.74) | 1.45 (1.17-1.81) |

**OR = odds ratio; CI = confidence interval; CCI = Charlson comorbidity index; ER = emergency room; COPD = chronic obstructive pulmonary disease*

**Additional file Table 3d** - Model performance measures for both the hospital outpatient visit - and hospital admission model predicting Persistent High-Cost status using a different High-Cost threshold (top-5%).

|  | **Hospital outpatient visit model** | **Hospital admission model** |
| --- | --- | --- |
|  | **Full model using top 5% High-Cost threshold** | **Full model using top 5% High-Cost threshold** |
| C-statistic  (Development cohort) | 0.81 | 0.78 |
| C-statistic  (Validation cohort) | 0.78 | 0.77 |
| Intercept  (Validation cohort) | -0.001 | -0.012 |
| Slope  (Validation cohort) | 1.052 | 1.032 |

*C-statistic = a measure of discrimination which indicates the model’s ability to discriminate between those with and those without the outcome. C-statistic ≥ 0.7 indicates good discrimination; Intercept = calibration intercept (0 indicates perfect calibration); Slope = calibration slope (1 indicates perfect calibration).*
