# Supplementary material for: Identifying persistent high-cost patients in the hospital for care management: development and validation of prediction models
Source: BMC Health Serv Res. 2024 Nov 26;24:1469. doi: 10.1186/s12913-024-11936-7 (PMC11590622; doi:10.1186/s12913-024-11936-7)
Supplement: Supplementary file 5 — Additional file 5. Predictive probabilities of the hospital outpatient visit - and hospital admission model for different cut-off points. [file 12913_2024_11936_MOESM5_ESM.docx]

**Additional file** **5** Predictive probabilities of the hospital outpatient visit - and hospital admission model for different cut-off points

|  | **Hospital outpatient visit model** | | | | | | **Hospital admission model** | | | | | |
| --- | --- | --- | --- | --- | --- | --- | --- | --- | --- | --- | --- | --- |
|  | **Full** | | | **Parsimonious^†^** | | | **Full** | | | **Parsimonious^†^** | | |
| Risk  cut-off | Sens (%) | Spec (%) | PPV (%) | Sens (%) | Spec (%) | PPV (%) | Sens (%) | Spec (%) | PPV (%) | Sens (%) | Spec (%) | PPV (%) |
| 5% | 18.8 | 97.2 | 9.3 | 16.5 | 97.6 | 9.4 | 63.4 | 74.2 | 11.5 | 64.7 | 70.6 | 10.4 |
| 10% | 9.8 | 99.1 | 13.8 | 8.5 | 99.1 | 13.1 | 31.0 | 92.2 | 17.3 | 33.1 | 91.4 | 16.8 |
| 15% | 5.7 | 99.6 | 18.8 | 5.1 | 99.6 | 17.4 | 20.4 | 96.5 | 23.7 | 19.3 | 96.0 | 20.4 |
| 20% | 3.2 | 99.7 | 19.3 | 2.4 | 99.8 | 20.7 | 12.7 | 98.3 | 27.7 | 13.4 | 97.7 | 23.6 |
| 25% | 2.0 | 99.8 | 18.0 | 1.3 | 99.9 | 20.3 | 8.4 | 99.0 | 31.1 | 9.8 | 98.8 | 29.6 |
| 30% | 1.3 | 99.8 | 18.4 | 1.0 | 99.9 | 20.0 | 5.7 | 99.4 | 33.7 | 6.4 | 99.3 | 32.7 |
| 35% | 0.8 | 99.9 | 18.6 | 0.6 | 99.9 | 20.0 | 4.3 | 99.7 | 40.9 | 4.5 | 99.6 | 35.8 |
| 40% | 0.5 | 99.9 | 15.8 | 0.3 | 99.9 | 20.0 | 2.9 | 99.8 | 42.3 | 2.8 | 99.7 | 35.9 |
| 45% | 0.4 | 99.9 | 17.2 | 0.2 | 100.0 | 21.5 | 2.1 | 99.9 | 45.8 | 2.3 | 99.8 | 42.1 |
| 50% | 0.2 | 99.9 | 13.6 | 0.2 | 100.0 | 22.2 | 1.5 | 99.9 | 43.2 | 1.7 | 99.9 | 45.0 |

*Sens = sensitivity; spec = specificity; PPV = positive predictive value. † parsimonious = counterpart to full model with the lowest number of relevant predictors yet comparable performance.*
